# The dietary bioflavonoid quercetin synergizes with epigallocathechin gallate (EGCG) to inhibit prostate cancer stem cell characteristics, invasion, migration and epithelial-mesenchymal transition

**DOI:** 10.1186/1750-2187-5-14

**Published:** 2010-08-18

**Authors:** Su-Ni Tang, Chandan Singh, Dara Nall, Daniel Meeker, Sharmila Shankar, Rakesh K Srivastava

**Affiliations:** 1 Department of Pharmacology, Toxicology and Therapeutics, and Medicine, The University of Kansas Cancer Center, The University of Kansas Medical Center, 3901 Rainbow Boulevard, Kansas City, KS, 66160, USA; 2 Department of Pathology and Laboratory Medicine, The University of Kansas Cancer Center, The University of Kansas Medical Center, 3901 Rainbow Boulevard, Kansas City, KS, 66160, USA

## Abstract

**Background:**

Much attention has been recently focused on the role of cancer stem cells (CSCs) in the initiation and progression of solid malignancies. Since CSCs are able to proliferate and self-renew extensively due to their ability to express anti-apoptotic and drug resistant proteins, thus sustaining tumor growth. Therefore, the strategy to eradicate CSCs might have significant clinical implications. The objectives of this study were to examine the molecular mechanisms by which epigallocathechin gallate (EGCG) inhibits stem cell characteristics of prostate CSCs, and synergizes with quercetin, a major polyphenol and flavonoid commonly detected in many fruits and vegetables.

**Results:**

Our data indicate that human prostate cancer cell lines contain a small population of CD44^+^CD133^+^ cancer stem cells and their self-renewal capacity is inhibited by EGCG. Furthermore, EGCG inhibits the self-renewal capacity of CD44^+^α2β1^+^CD133^+ ^CSCs isolated from human primary prostate tumors, as measured by spheroid formation in suspension. EGCG induces apoptosis by activating capase-3/7 and inhibiting the expression of Bcl-2, survivin and XIAP in CSCs. Furthermore, EGCG inhibits epithelial-mesenchymal transition by inhibiting the expression of vimentin, slug, snail and nuclear β-catenin, and the activity of LEF-1/TCF responsive reporter, and also retards CSC's migration and invasion, suggesting the blockade of signaling involved in early metastasis. Interestingly, quercetin synergizes with EGCG in inhibiting the self-renewal properties of prostate CSCs, inducing apoptosis, and blocking CSC's migration and invasion. These data suggest that EGCG either alone or in combination with quercetin can eliminate cancer stem cell-characteristics.

**Conclusion:**

Since carcinogenesis is a complex process, combination of bioactive dietary agents with complementary activities will be beneficial for prostate cancer prevention and/ortreatment.

## Background

Prostate cancer currently accounts for 29 percent of all new cancer diagnoses in men. It is estimated that more than 27,000 U.S. men died of this disease in 2009, and this incidence is likely to increase as the male population ages [1,2]. The development and progression of prostate cancer is a slow and complex process that involves multiple steps of tumorigenic transformation differentially modulated by endocrine, nutritional and perhaps inflammatory/immune factors. To date, the molecular mechanisms that mediate the initiation and progression of prostate cancer remain poorly understood. Preventive strategies for prostate cancer require considerable new knowledge about the mechanisms underlying the pathogenesis and progression of the disease. Cancer stem cells (CSCs) are generally thought of as self-renewing cells that are able to reinitiate a tumor for several generations and can give rise to a spectrum of differentiated cells [3-5]. CSCs, like normal stem cells, are also more likely to express antiapoptotic and drug-resistance genes, making them impervious to most anticancer therapeutics [6-13]. In order to eradicate a tumor and prevent recurrence, it is imperative that cancer stem cells be specifically targeted.

Cancer stem/progenitor cells may exhibit characteristics similar to normal stem cells. CSCs have limitless potential for self-renewal and can efficiently form tumors in immunodeficient mice that recapitulate the heterogeneity observed in original tumors [14-17]. Recently, CSCs have been described in several human tumors including breast, gastrointestinal, lung, prostate, brain, and melanoma on the basis of their clonogenic efficiency *in vitro *and ultimately tumorigenicity *in vivo *[15,16,18-21]. The identification and characterization of CSCs might have enormous clinical implications: for instance, it is has been shown that CSCs might survive chemo- as well as radiotherapy [12,22-31], due to the preferential expression of resistance molecules or activation of specific signaling pathways. Therefore, understanding the mechanisms of drug resistance and development of novel strategies to kill CSCs are urgently needed.

Human prostate epithelial (HPE) exhibit stem cell characteristics, expressing embryonic stem cell markers, including Oct-4, Nanog, and Sox-2, in addition to the early progenitor cell markers CD133, CD44, and nestin. HPET cells do not express p63 and AR, similar to other reports on prostate stem cells [32,33]. Most importantly, clonally derived HPET cells are capable of reconstituting the original prostate tumor from which they were derived and retain the ability to differentiate into basal, luminal, and neuroendocrine epithelial cell types of the prostate *in vivo*. CD44 is a basal cell marker that has been studied as a marker for human prostate CSCs [34,35].

Examination of human prostate cancer cell lines and xenografts indicate that the CD44^+ ^population is more proliferative, clonogenic, tumorigenic, and metastatic than CD44^- ^cells. Prostate cancer cell lines sorted for high expression of CD44 have been associated with enhanced expression of "stemness" markers including BMI, β-catenin, SMO, and Oct 3/4 [36-38]. Moreover, CD44^+^α2β1+CD133^+ ^subpopulations obtained from human tissue have enhanced capacity for *in vitro *serial passaging.

Epithelial-mesenchymal transition (EMT) induction in cancer cells results in the acquisition of invasive and metastatic properties [13,29,39-41]. Recent reports indicate that the emergence of CSCs occurs in part as a result of EMT, for example, through cues from tumor stromal components. CSCs and EMT-type cells, which shares molecular characteristics with CSCs, have been believed to play critical roles in drug resistance and early cancer metastasis as demonstrated in several human malignancies including prostate cancer [13,29,39-41]. Thus, the discovery of molecular knowledge of drug resistance and metastasis in relation to CSCs and EMT in prostate cancer is becoming an important area of research, and such knowledge is likely to be helpful in the discovery of newer drugs as well as designing novel therapeutic strategies for the treatment of prostate cancer with better outcome.

Epidemiological and dietary intervention studies in animals and humans have suggested that diet-derived phenolics, in particular the flavonoids, may play a beneficial role in inhibiting, reversing or retarding tumorigenesis in many types of cancers, including prostate cancer [42-47]. Flavonoids are known to possess anti-inflammatory, anti-oxidant, anti-allergic, hepato-protective, anti-thrombotic, anti-viral and anti-carcinogenic activities [42-47]. These activities of flavonoids are thought to be mediated by interfering with a large number of mammalian enzymes, such as detoxifying enzymes that are involved in cell division and proliferation pathways [42,45,48-50]. Among the flavonoids, quercetin (3,3',4',5,7-pentahydroxyflavone) is a naturally occurring flavones, and is a component of most edible fruits and vegetables, with the highest concentrations being found in onions, apples, and red wine [42,45,51]. Several studies have shown that quercetin has a broad range of pharmacological properties that include antioxidant and anti-inflammatory activities [52]. Quercetin treatment has been associated with selective antiproliferative effects and induction of cell death, predominantly through an apoptotic mechanism, in cancer cell lines but not in normal cells [53-56]. The anti-proliferative effect of quercetin is believed to be exerted by producing arrest in the G_1 _phase of the cell cycle or through interaction with cell cycle-regulated proteins, like cyclin D1 and CDK4 [57-59]. Quercetin also has been shown to induce apoptosis by releasing cytochrome *c *and activating caspase-9 and caspase-3 [60-62]. Moreover, quercetin is believed to be a potential PI3K inhibitor, an enzyme involved in the pivotal cell survival pathway [63,64]. Interestingly, quercetin has been shown to enhance the anticancer activities of several chemotherapeutic drugs and inhibit the expression of P-glycoprotein [65-70]. Therefore, the ability of quercetin to prevent and/or to retard tumor growth is probably a multifunctional effect. Although the underlying mechanisms governing these effects are not yet fully understood, the available evidence collectively indicates that quercetin may be of therapeutic benefit in clinical settings, suggesting its potential use as an anticancer agent or an adjunct to current cancer therapies.

Epigallocathechin gallate (EGCG) from tea has gained recognition as important chemopreventive agent and as modulators of tumor cell response to chemotherapy [71-75]. EGCG downregulates Pg-P and BCRP in a tamoxifen resistant MCF-7 cell line [76]. *In vitro *and *in vivo *studies have demonstrated that EGCG affect a wide array of molecular pathways, resulting in inhibition of cell growth, invasion, angiogenesis, and metastasis [77,78]. In prostate cancer, EGCG alter numerous intracellular pathways, including inhibition of ERK1/2- and Akt-mediated signaling, inhibition of PMA-dependent PKC activation, alteration of Bcl-2 family members ratio, and activation of caspases [79-83]. We have recently demonstrated that EGCG caused growth arrest at G1 stage of cell cycle through regulation of cyclin D1, cdk4, cdk6, p21/WAF1/CIP1 and p27/KIP1, and induced apoptosis through generation of reactive oxygen species and activation of caspase-3 and caspase-9 [77,78,84]. EGCG inhibited expressions of Bcl-2 and Bcl-X_L _and induced expressions of Bax, Bak, Bcl-X_S _and PUMA [77,78,84]. Furthermore, the activities of Ras, Raf-1 and ERK1/2 were inhibited, whereas the activities of MEKK1, JNK1/2 and p38 MAP kinases were induced by EGCG. Inhibition of cRaf-1 or ERK enhanced EGCG-induced apoptosis, whereas inhibition of JNK or p38 MAP kinase inhibited EGCG-induced apoptosis. EGCG inhibited the activation of p90 ribosomal protein S6 kinase, and induced the activation of cJUN [77,78,84]. Xenograft and TRAMP models have shown that green tea or EGCG can decrease the tumorigenic potential of prostate cancer (7-10). These studies demonstrate that EGCG induces growth arrest and apoptosis through multiple mechanisms. Overall, these findings suggest that green tea and its constituents can be used for chemoprevention to target cancer stem cells.

The objectives of our study were to characterize prostate cancer stem cells, and examine the molecular mechanisms by which quercetin enhances the inhibitory effects of EGCG on self-renewal capacity of prostate cancer stem cells.

## Results

### CD44+ and CD133+ CSCs can be isolated from PC-3 and LNCaP cells

**Figure 1:**
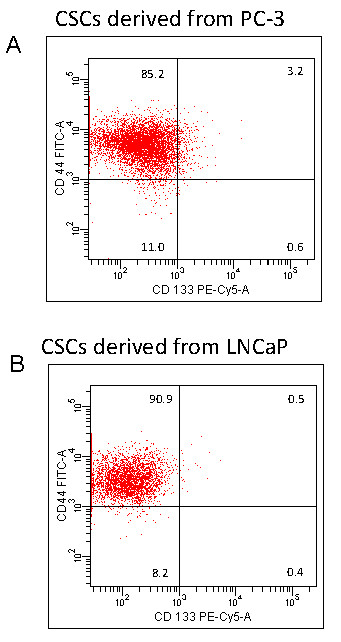
**The presence of CSCs in PC-3 and LNCaP cells**. (A), PC-3 cells were harvested, and stained with anti-CD44-FITC, anti-CD133-PE or isotype control antibody. The presence of CD44+ and CD133+ cells were examined by the flowcytometry. (B), LNCaP cells were harvested, and stained with anti-CD44-FITC, anti-CD133-PE or isotype control antibody. The presence of CD44+ and CD133+ cells were examined by the flowcytometry.

The existence of stem-like cells in culture and xenografted tumors has been demonstrated [85-87]. Therefore, we first examined the existence of CD44+ and CD133+ CSCs in prostate cancer cell lines by flow cytometry (Fig 1). Our data demonstrated the existence of 3.2% and 0.5% CD44+ plus CD133+ CSCs in both PC-3 and LNCaP cells, respectively. These results indicate the possible presence of stem-like cells in PC-3 and LNCaP cells.

### EGCG inhibits the growth of cancer stem cells isolated from human prostate cancer cell lines

**Figure 2:**
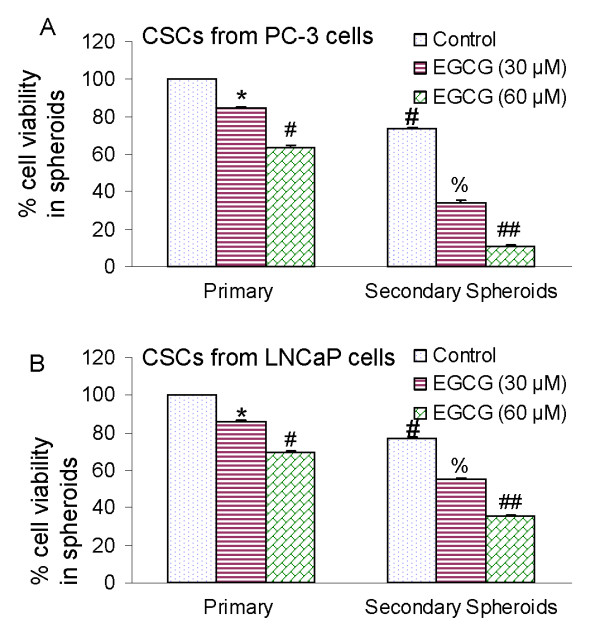
**Effects of EGCG on spheroid cell viability in cancer stem cells (CSCs) derived from human prostate cancer cell lines**. (A), The CSCs were enriched from PC-3 cells, and grown in suspension in keratinocyte serum-free medium supplemented with B27, 10 ng/ml EGF, and 10 ng/ml basic fibroblast growth factor (Invitrogen). Prostate CSCs were re-seeded in suspension and treated with EGCG (0-60 μM) for 7 days. The spheroids were dissociated with Accutase (Innovative Cell Technologies, Inc.), and sieved through a 40-μm filter. Cell viability was measured by trypan blue assay. For secondary sphere formation, CSCs were reseeded and treated with EGCG for 7 days. Data represent mean ± SD. *, #, % or ## = significantly different from control, P < 0.05. (B), Prostate cancer stem cells were isolated from LNCaP cells, seeded in suspension and treated with EGCG (0-60 μM) for 7 days. At the end of incubation period, sheroids were dissociated with Accutase (Innovative Cell Technologies, Inc.), and sieved through a 40-μm filter. Cell viability was measured by trypan blue assay. Data represent mean ± SD. *, #, % or ## = significantly different from control, P < 0.05.

Since CSCs has been successfully isolated from established human cancer cells lines, we examined the effects of EGCG on cancer stem cells (CD44^+^CD133^+^) isolated from human prostate cancer cell lines (Fig. 2). Isolated CSCs were grown in pancreatic cancer stem cell medium in suspension and treated with various doses SFN (0-10 μM) for 7 days. At the end of incubation period, spheroids were harvested, resuspended, and cell viability was measured. EGCG inhibited viability of prostate CSCs isolated from PC-3 and LNCaP cell lines in a dose-dependent manner. These data suggest that human prostate cancer cell lines possess a small population of CSCs which are responsive to EGCG treatment.

### EGCG inhibits the formation of primary and secondary tumor spheroids and cell viability of human prostate cancer stem cells

**Figure 3:**
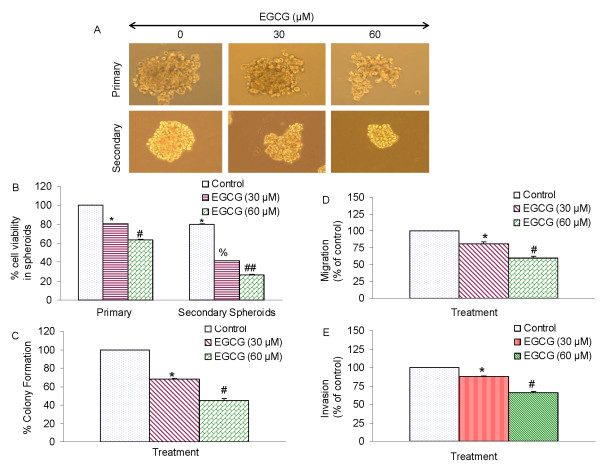
**Effects of EGCG on tumor spheroids and cell viability of prostate cancer stem cells (CSCs)**. (A), Prostate CSCs were seeded in suspension and treated with EGCG (0-60 μM) for 7 days. Pictures of spheroids formed in suspension were taken by a microscope. (B), Prostate CSCs were seeded in suspension and treated with EGCG (0-60 μM) for 7 days. At the end of incubation period, all the spheroids were collected and resuspended. Cell viability was measured by trypan blue assay. Data represent mean ± SD. *, #, % or ## = significantly different from control, P < 0.05. (c), EGCG inhibits colony formation by prostate CSCs. Prostate CSCs were seeded in soft agar and treated with various doses of EGCG and incubated at 4°C for 21 days. At the end of incubation period, colonies were counted. Data represent mean ± SD. * or # = significantly different from respective controls, P < 0.05. (D), Transwell migration assay. Prostate CSCs were plated in the top chamber of the transwell and treated with EGCG (0-60 μM) for 24 h. Cells migrated to the lower chambered were fixed with methanol, stained with crystal violet and counted. Data represent mean ± SD. * or # = significantly different from respective controls, P < 0.05. (E) Matrigel invasion assay. Prostate CSCs were plated onto the Matrigel-coated membrane in the top chamber of the transwell and treated with EGCG (0-60 μM) for 48 h. Cells invaded to the lower chambered were fixed with methanol, stained with crystal violet and counted. Data represent mean ± SD. * or # = significantly different from respective controls, P < 0.05.

Since EGCG inhibited the growth of CSCs isolated from established prostate cancer cell lines, we sought to examine whether EGCG could also inhibit the growth of CSCs isolated from human primary prostate tumors. We first examined the effects of EGCG on the CSC growth by measuring spheroid formation and cell viability in prostate CSCs expressing CD44^+^α2β1+CD133^+^. CSCs were grown in prostate cancer stem cell defined medium in suspension, and treated with EGCG for 7 days. At the end of incubation period, spheroids in each well were photographed. EGCG inhibited the growth of spheroids in suspension in a dose-dependent manner (Fig. 3A). The spheroids from each treatment group were collected and resuspended for counting cell viability. EGCG inhibited prostate CSC viability in a dose-dependent manner (Fig. 3B).

Since EGCG inhibited the growth of tumor spheroid and cell viability of CSCs, we next sought to examine the effects of EGCG on colony formation. Prostate CSCs were grown in agar, and treated with various doses of EGCG for 3 weeks. At the end of incubation period, colonies were counted. EGCG inhibited the growth of colonies in a dose-dependent manner (Fig. 3C). These data suggest that EGCG can be effective in inhibiting the self-renewal capacity of prostate cancer stem cells.

Since CSCs appear to play a significant role in early metastasis [13,41,88,89], we sought to measure the effects of EGCG on invasion and migration of CSCs. EGCG inhibited cell migration and invasion (Fig. 3D and 3E). These data suggest that EGCG can be a useful agent in targeting prostate cancer stem cells.

### Inhibition of Nanog enhances the effects of EGCG on spheroid formation by human prostate cancer stem cells

**Figure 4:**
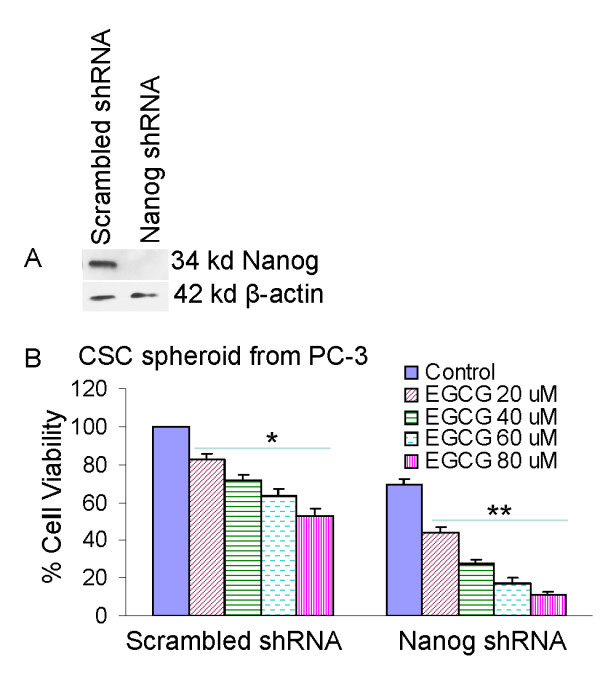
**Inhibition of Nanog enhances the effects of EGCG on CSC spheroid formation**. CD133+ and CD44+ CSCs were isolated from PC-3 cells and plated in six-well ultralow attached plates at a density of 1,000 cells/ml. (A), CSCs were transduced with either scrambled shRNA or Nanog shRNA expressing lentiviral vector (pLKO.1), and cell lysates were collected and western blot analysis was performed using anti-Nanog antibody. (B), CSC/scrambled and CSC/Nanog shRNA were seeded as described above and treated with EGCG (0-80 μM). After 7 days, spheroids were collected and cell suspentions were prepared and viable cells were counted by trypan blue assay. Data represent mean ± SD. * or ** = significantly different from control, P < 0.05.

A high level of Nanog is a key regulator of embryonic stem cell (ESC) self-renewal and puripotency [90,91]. Nanog-deficient ES cells and embryos lose their pluripotency [92]. Since pluripotent transcription factor Nanog is highly expressed in CSCs compared to normal cells [93-97], we examined the effects of inhibiting Nanog on antiproliferative effects of EGCG in human prostate CSCs expressing (CD44^+^α2β1^+^CD133^+^). Lentiviral mediated transduction of Nanog shRNA inhibited Nanog protein expression (Fig. 4). EGCG inhibited stem cell viability in CSC spheroids transduced with Nanog-scrambled shRNA in a dose-dependent manner. The inhibition of Nanog by shRNA further enhanced the antiproliferative effects of EGCG on prostate CSCs. These data suggest that inhibition of Nanog may be an attractive target to enhance the anticancer activities of EGCG in CSCs.

### EGCG inhibits the expression of XIAP and Bcl-2 and induces caspase-3 activation in human prostate cancer stem cells

**Figure 5:**
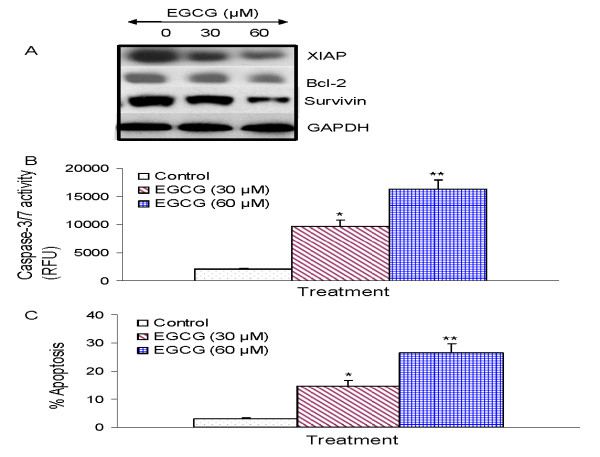
**Regulation of apoptosis-related proteins, caspase-3/7 activity and apoptosis by EGCG on CSCs derived from human primary prostate tumors**. (A), Regulation of apoptosis-related proteins. Prostate CSCs from primary tumors were treated with EGCG (0-60 μM) for 48 h. The Western blot analyses were performed to examine the expression of XIAP, Bcl-2 and survivin, and GAPDH. (B), Regulation of caspase-3/7 activity by EGCG. Prostate CSCs were treated with EGCG (0-60 μM) for 24 h, and caspase-3/7 activity was measured as per manufacturer's instructions. Data represent mean ± SD. * or ** = significantly different from control, P < 0.05. (C), Regulation of apoptosis by EGCG. Prostate CSCs were treated with EGCG (0-60 μM) for 48 h, and apoptosis was measured by TUNEL assay. Data represent mean ± SD. * or ** = significantly different from control, P < 0.05.

Since members of the IAP and Bcl-2 play important roles in cell survival and apoptosis [98,99], we sought to examine the effects of EGCG on the expression of XIAP, Bcl-2 and survivin, activation of caspase-3/7 and induction of apoptosis in prostate CSCs (Fig. 5). EGCG inhibited the expression of XIAP, Bcl-2 and survivin in prostate CSCs (Fig. 5A). Furthermore, EGCG induced the activation of caspase-3/7 and apoptosis in prostate CSCs in a dose-dependent manner (Fig. 5B and 5C). These data suggest that EGCG can induce apoptosis in CSCs by engaging cell-intrinsic pathway of apoptosis.

### EGCG inhibits the expression of epithelial-mesenchymal transition marker (EMT) in human prostate cancer stem cells

**Figure 6:**
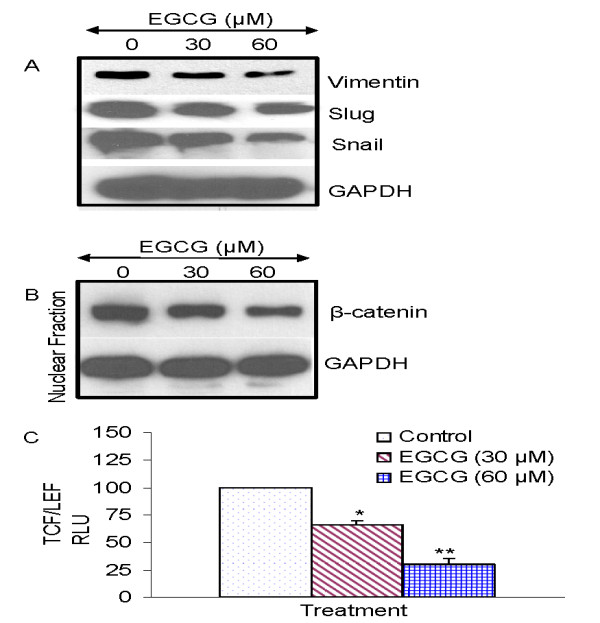
**Regulation of epithelial mesenchymal transition factors by EGCG in prostate cancer stem cells isolated from primary tumors**. **(A)**, Prostate CSCs were treated with EGCG (0-60 μM) for 48 h. At the end of incubation period, the expression of vimentin, slug, and snail was measured by the Western blot analysis. (B), Effects of EGCG on the expression of nuclear β-catenin. Prostate CSCs were treated with EGCG (0-60 μM) for 48 h. At the end of incubation period, cells were harvested and nuclear fractions were prepared. The expression of β-catenin and GAPDH was measured by was measured by the Western blot analysis. (c), Effects of EGCG on TCF-1/LEF activity. Prostate CSCs were transduced with lentiviral Top-dGFP-reporter (pRLR.sm-18.ppt). Transduced CSCs were treated with EGCG (0-60) for 3 days and the GFP fluorescence was measured. Data represent mean ± SD. * or ** = significantly different from control, P < 0.05.

During cancer metastasis, the mobility and invasiveness of cancer cells increase. To detach from neighboring cells and invade adjacent cell layers, carcinoma cells must lose cell-cell adhesion and acquire motility. The highly conserved EMT program has been implicated in dissemination of carcinoma cells from primary epithelial tumors [100]. Tumor progression is frequently associated with the downregulation of E-cadherin [100], and upregulation of vimentin and several transcription factors including Snail, Twist and Slug [101-103]. Cancer stem cells undergoing metastasis usually express EMT markers. We therefore examined the regulation of EMT markers by EGCG. As expected, EGCG inhibited the expression of vimentin, slug and snail (Fig. 6A). EGCG also inhibited the expression of nuclear β-catenin in prostate CSCs (Fig. 6B). We next examined the effects of EGCG on TCF-1/LEF activity by reporter assay. As shown in Fig. 6C, EGCG inhibited TCF-1/LEF activity in prostate CSCs. These data suggest that inhibition of EMT markers by EGCG could inhibit early metastasis of cancer stem cells.

### Quercetin enhances the effects of EGCG on cell viability in spheroids, colony formation, apoptosis, migration and invasion by prostate cancer stem cells

**Figure 7:**
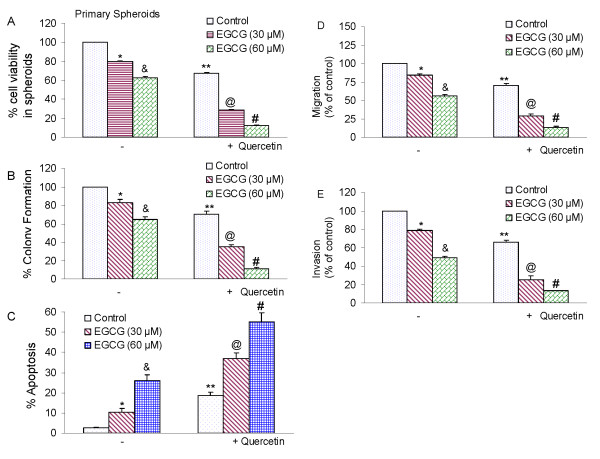
**Quercetin synergizes with EGCG to inhibit self-renewal capacity of prostate cancer CSCs isolated from primary tumors**. (A), Quercetin synergizes with EGCG to inhibit spheroid cell viability. Prostate CSCs were seeded in suspension and treated with EGCG (0-60 μM) with or without quercetin (20 μM) for 7 days. At the end of incubation period, all the spheroids were collected and resuspended. Cell viability was measured by trypan blue assay. Data represent mean ± SD. *, &, **, @ or # = significantly different from control, P < 0.05. (B), Quercetin synergizes with EGCG to inhibit colony formation. Prostate CSCs were seeded in soft agar and treated with various doses of EGCG (0-60 μM) with or without quercetin (20 μM) and incubated at 4°C for 21 days. At the end of incubation period, colonies were counted. Data represent mean ± SD. *, &, **, @ or # = significantly different from control, P < 0.05. (C), Quercetin synergizes with EGCG to induce apoptosis. Prostate CSCs were seeded in suspension and treated with EGCG (0-60 μM) with or without quercetin (20 μM) for 7 days. At the end of incubation period, all the spheroids were collected. Apoptosis was measured by TUNEL assay. Data represent mean ± SD. *, &, **, @ or # = significantly different from control, P < 0.05. (D), Migration assay. Prostate CSCs were plated in the top chamber of the transwell and treated with EGCG (0-60 μM) with or without quercetin (20 μM) for 24 h. Cells migrated to the lower chambered were fixed with methanol, stained with crystal violet and counted. Data represent mean ± SD. *, &, **, @ or # = significantly different from control, P < 0.05. (E) Matrigel invasion assay. Prostate CSCs were plated onto the Matrigel-coated membrane in the top chamber of the transwell and treated with EGCG (0-60 μM) with or without quercetin (20 μM) for 48 h. Cells invaded to the lower chambered were fixed with methanol, stained with crystal violet and counted. Data represent mean ± SD. *, &, **, @ or # = significantly different from control, P < 0.05.

Quercetin has been shown to enhance the effects of anticancer drugs and sensitize cancer cells to chemotherapy [65,67,70,104-106]. We therefore examined whether quercetin enhances the inhibitory effects of EGCG on self-renewal, migration and invasion capacities of prostate CSCs (Fig. 7). EGCG inhibited cell viability in spheroids, colony formation, migration and invasion by CSCs in a dose-dependent manner (Fig. 7A,B,D and 7E). Quercetin, although effective alone, further enhanced the biological effects of EGCG on cell viability, colony formation, migration and invasion. Furthermore, EGCG and quercetin alone induced apoptosis (Fig. 7C). Interestingly, quercetin synergizes with EGCG to induce apoptosis in prostate CSCs. These data suggest that quercetin can be used with EGCG to selectively target prostate CSCs.

## Discussion

Our study demonstrates, for the first time, that cancer preventive effects of EGCG and quercetin can inhibit the self-renewal capacity of prostate cancer stem cells. Our data are agreement with others who have demonstrated that prostate cancer cell lines contain a small population of cells having stem cell characteristics. EGCG can synergies with quercetin to inhibit the self-renewal capacity of prostate CSCs expressing CD44^+^α2β1^+^CD133^+^. EGCG induces apoptosis by activating capase-3/7, inhibiting Bcl-2, survivin and XIAP. EGCG inhibits the expression of vimentin, slug, snail and nuclear β-catenin which were accompanied by the inhibition of TCF-1/LEF reporter activity. The inhibition of EMT-related genes by EGCG suggests the blockade of signaling involved in early metastasis. Interestingly, quercetin synergizes with EGCG in inhibiting self-renewal capacity of prostate CSCs and their migration and invasion. These data suggest that EGCG either alone or in combination with quercetin can eliminate cancer stem cell-characteristics. Since carcinogenesis is a complex process, combination of bioactive dietary agents with complementary activities will be beneficial for prostate cancer prevention and/or treatment.

Most carcinomas comprise of a heterogeneous cell population with marked differences in their ability to proliferate and differentiate as well as their ability to reconstitute the tumor upon transplantation. Recent studies indicate the presence of a small, stem-like cell population in several human cancers that is crucial for the tumor repopulation. In a recent study, it has been demonstrate that five out of six prostate cancer cell lines formed clear holo-, mero-, and paraclones [87]. DU145 holoclones were maintained in culture for several passages, which is indicative of self-renewal ability. A small fraction (0.01%) of CD133^+ ^cells was detected in DU145 cell lines [87]. CD133^+ ^cells; however, like DU145 BCRP^+ ^(0.15%) cells, they were not more clonogenic, and they did not show more holoclone formation than the marker-negative cells or unselected cells. Immunohistochemistry revealed alpha2-integrin and BCRP as potential stem cell markers and CK5 with the combination of CK18 to distinguish transient amplifying cells [87]. Similarly, in the present study, the proportions of CD133^+ ^cells in PC-3 and LNCaP cell lines were 3.8% and 0.9%, respectively; and these cells were able to form primary and secondary spheres. This led to the hypothesis that the entire population of tumor cells might arise from a rare subpopulation of putative cancer stem/progenitor-like cells, also known as tumor-initiating cells or cancer stem cells [20,87,107,108]. CSCs share principle characteristics with adult stem cells namely self-renewal, high proliferative potential, clonogenicity, and multipotency. In addition, they have the ability to reproducibly form the same tumor phenotype as in the patient and to undergo differentiation into non-tumorigenic cells [87,109,110]. TICs were first isolated from patients with hematologic malignancies in which a few cells could initiate a new tumor [111]. During the past few years, CSCs were also identified and isolated from solid tumors such as breast, brain, colon, pancreatic, and prostate tumors [14,15,112-117]. The cancer stem cell hypothesis has provided a paradigm shift in our understanding of carcinogenesis, metastasis, and tumor biology. The identification of CSCs has important implications in the way cancer treatment should be conceived and future therapeutic approaches will be designed. Whether the subpopulation of CSCs is involved in the formation of distant metastases, tumor dormancy and therapy resistance has remained poorly understood.

The molecular mechanism by which CSCs (and the supportive stroma) play roles in the formation of distant metastases has remained largely elusive. The development of more effective cancer therapies in advanced prostate cancer may, thus, benefit from the outcome of new studies and require selective targeting of this specific subpopulation of metastasis-initiating cells. In prostate cancer, innovative studies have led to the identification of CD44+/α2β1high/CD133+ prostate cancer stem cells [20,118]. Because of the observed heterogeneity in prostate cancer, the use of single-cell markers for the selection, characterization/identification, and functional evaluation of stem/progenitor-like prostate cancer cells has been a major impediment and the reliability of cell surface markers such as CD133 as the sole way to isolate TICs remains controversial to date [108,119]. Deregulation of ALDH enzyme activity is implicated in the pathophysiology of various hematologic and epithelial cancers [120]. The introduction of FACS-based viable cell sorting for ALDH activity (ALDEFLUOR assays) in tumor biology has further substantiated a role of ALDH^hi ^subpopulations of cancer cells in carcinogenesis [114,121-123]. High ALDH activity, as detected by the ALDEFLUOR assay, can thus be used as a functional marker to isolate TICs in several types of epithelial cancers, including those of breast, lung and colon [114,121-125]. A recent study has demonstrated that high ALDH activity can be used to isolate human prostate cancer cells with significantly enhanced clonogenic and migratory properties in vitro as well as elevated tumor- and metastasis-initiating abilities *in vivo *[126]. The percentage of ALDH^hi^ cells in prostate cancer cell lines also seems to be related to tumorigenicity and metastatic behavior.

Quercetin is a ubiquitous bioactive plant flavonoid that has been shown to inhibit cell proliferation in several cancer types. Quercetin has been shown to decrease Akt phosphorylation and survivin expression in prostate cancer cells [64,127]. Studies investigating molecular mechanisms that underlie the inhibition of cell proliferation by quercetin demonstrated that a treatment including quercetin triggered numerous cellular events including DR5 upregulation [106,128-130], p53 activation [131-134], cell cycle arrest [58,59], and induction of caspase-mediated apoptosis in cancer cells. Quercetin down-regulates the expression of Hsp90 which, in turn, induces growth inhibition and apoptosis in prostate cancer cells while exerting no quantifiable effect on normal prostate epithelial cells. Quercetin has also been shown to inhibit the expression of BCRP and MDR expression [66,135,136]. In our study, quercetin has been able to synergies with EGCG in inhibiting the self-renewal capacity of prostate CSCs, migration and invasion, and inducing apoptosis. Since quercetin is a MDR modulator, it can sensitize CSCs to anticancer drugs.

Recent evidence suggests a shared genomic fingerprint between embryonic stem cells, cancer cells, and cancer stem cells. Activation targets of Nanog, Oct4, Sox2 and c-Myc are more frequently overexpressed in certain tumors. In the absence of bona fide cancer stem cell lines, human embryonic stem cells, which have similar properties to cancer and cancer stem cells, have been an excellent model throwing light on the anticancer affects of various putative anticancer agents. Nanog, Sox2 and Oct4 are transcription factors which are essential to maintaining the pluripotent embryonic stem cell phenotype. Oct4 and Sox2 bind to the Nanog promoter in living mouse and human ESCs [137]. Nanog, Oct4 and Sox2 co-occupy and regulate their own promoters together with other developmental genes with diverse functions and collaborate to form an extensive regulatory circuitry including autoregulatory and feed-forward loops [137-139]. A high level of Nanog is a key regulator of ESC self-renewal and puripotency. Nanog-deficient ES cells and embryos lose their pluripotency [92]. Nanog overexpression leads to the clonal expansion of ES cells through circumvention of the LIF-dependent Stat-3 pathway and sustained Oct-4 expression levels [92,140]. Genome-wide gene expression profiling shows that Nanog is expressed at high levels in testicular carcinoma in situ and germ cell tumors [141]. Positive correlations of Oct4, Nanog, or CD133 expression on tumor stage were shown on oral squamous cell carcinoma patient tissues [142]. In our study, inhibition of Nanog by shRNA enhanced the inhibitory effects of EGCG on tumor sphere formation and cell viability, suggesting its requirement for self-renewal of CSCs. Medulloblastoma-associated CSCs selected by serum-free medium with bFGF and EGF can form 3 D spheroids and display enhanced self-renewal and highly co-expressed stem cell genes (Oct4, Nanog, Nestin, and Musashi-1) as well as anti-apoptotic genes (Bcl-2 and Bcl-X_L_) [143]. These finding suggest that inhibition of Nanog and/or other pluripotent factors could be a novel strategy to kill CSCs.

EMT is an embryonic program in which epithelial cells lose their characteristics and gain mesenchymal features. Therefore, EMT might play a very important role during malignant tumor progression. Accumulating evidence suggest that transformed epithelial cells can activate embryonic programs of epithelial plasticity and switch from a sessile, epithelial phenotype to a motile, mesenchymal phenotype. Induction of EMT can, therefore, lead to invasion of surrounding stroma, intravasation, dissemination and colonization of distant sites. Under the cancer stem cell hypothesis, sustained metastatic growth requires the dissemination of a CSC from the primary tumor followed by its re-establishment in a secondary site. In the present study, EGCG inhibited the expression of EMT markers (expression of vimentin, nuclear β-catenin and TCF-1/LEF reporter activity) and also inhibited the transcription factors slug and snail, which are required for induction of EMT. The inhibition of EMT markers by EGCG suggests that it could inhibit early metastasis of prostate CSCs. Furthermore, quercetin will further enhance the biological effects of EGCG in inhibiting EMT and hence early metastasis.

In conclusion, we have demonstrated that EGCG inhibited self-renewal capacity of prostate CSCs, and these properties of EGCG were further enhanced by quercetin. EGCG inhibited the expression of transcription factors which are required for maintaining stem-cell pluripotency. Inhibition of Nanog could be considered as a novel strategy to enhance the biological effects of anticancer and chemopreventive agents or sensitize those cells which are resistant to chemotherapy or irradiation. Moreover, EGCG inhibited expression of proteins involved in the EMT, suggesting the blockade of signaling involved in early metastasis. Furthermore, combination of quercetin with EGCG had synergistic inhibitory effects on self-renewal and metastatic properties of prostate CSCs. These data suggest that EGCG either alone or in combination with quercetin can be used for the prevention and/or treatment of prostate cancer. However, further studies are needed to validate the combination of EGCG and quercetin in an appropriate *in vivo *model.

## Methods

### Reagents

Antibodies against β-catenine, vimentin, slug, snail, GAPDH, XIAP, survivin, Bcl-2, and Nanog were purchased from Cell Signaling Technology, Inc. (Danvers, MA). EGCG and quercetin were purchased from LKT Laboratories, Inc. (St. Paul, MN). Enhanced chemiluminescence (ECL) Western blot detection reagents were from Amersham Life Sciences Inc. (Arlington Heights, IL). Terminal Deoxynucleotidyl Transferase Biotin-dUTP Nick End Labeling (TUNEL) assay kit was purchased from EMD Biosciences/Calbiochem (San Diego, CA). All other chemicals were purchased from Sigma-Aldrich (St Luis, MO).

### Cell Culture

PC-3 and LNCaP cells were obtained from the American Type Culture Collection (Manassas, VA). Human prostate cancer stem cells (CD44^+^α2β1^+^CD133^+^) were from Celprogen Inc. (San Pedro, CA). CSCs were cultured in DMEM supplemented with 1% N2 Supplement (Invitrogen), 2% B27 Supplement (Invitrogen), 20 ng/ml human platelet growth factor (Sigma-Aldrich), 100 ng/ml epidermal growth factor (Invitrogen) and 1% antibiotic-antimycotic (Invitrogen) at 37°C in a humidified atmosphere of 95% air and 5% CO_2_.

### Tumor Spheroid Assay

Spheroid forming assays were performed as described elsewhere [144,145]. In brief, single cells were plated in six-well ultralow attachment plates (Corning Inc., Corning, NY) at a density of 1,000 cells/ml in DMEM supplemented with 1% N2 Supplement (Invitrogen), 2% B27 Supplement (Invitrogen), 20 ng/ml human platelet growth factor (Sigma-Aldrich), 100 ng/ml epidermal growth factor (Invitrogen) and 1% antibiotic-antimycotic (Invitrogen) at 37°C in a humidified atmosphere of 95% air and 5% CO_2_. Spheroid were collected after 7 days and dissociated with Accutase (Innovative Cell Technologies, Inc.). The cells obtained from dissociation were sieved through a 40-μm filter, and counted by coulter counter using trypan blue dye.

### Soft agar colony assay

Cell suspensions (2,500 cells) were prepared using 0.4% Noble agarose (Becton Dickinson) and overlayed onto a 60-mm dish containing a solidified bottom layer of 0.6% agarose in medium. Once the top layer solidified, 1 ml of medium was placed on top of the cell layer. After treatment, plates were incubated for 3 weeks and colonies were counted by using microscopy.

### Transwell Migration assay

For transwell migration assays, 1 × 10^5 ^prostate CSCs were plated in the top chamber onto the noncoated membrane (24-well insert; pore size, 8 μm; Corning Costar) and allowed to migrate toward serum-containing medium in the lower chamber. Cells were fixed after 24 hours of incubation with methanol and stained with 0.1% crystal violet (2 mg/mL, Sigma-Aldrich). The number of cells invading through the membrane was counted under a light microscope (40×, three random fields per well).

### Transwell invasion assay

For invasion assay, 1 × 10^5 ^cells were plated in the top chamber onto the Matrigel coated Membrane (24-well insert; pore size, 8 μm; Corning Costar). Each well was coated freshly with Matrigel (60 μg; BD Bioscience) before the invasion assay. Cells were plated in medium without serum or growth factors, and medium supplemented with serum was used as a chemoattractant in the lower chamber. The cells were incubated for 48 hours and cells that did invade through the pores were removed by a cotton swab. Cells on the lower surface of the membrane were fixed with methanol and stained with crystal violet. The number of cells invading through the membrane was counted under a light microscope (40×, three random fields per well).

### Viral production and infection

Nanog shRNA construct (pLKO.1-puro, Mission RNAi) was from Open Biosystems. Lentiviral-TOP-dGFP-reporter **(**pRRL.sin-18.ppt) has been described elsewhere [146]. Lentivirus was produced by triple transfection of HEK 293T cells. Viral supernatants were collected and concentrated by ultracentrifugation to produce virus stocks with titers of 1 × 10^8 ^to 1 × 10^9 ^infectious units per milliliter. Viral supernatant was collected for three days by ultracentrifugation and concentrated 100-fold [147]. Titers were determined on HEK293T cells. Human prostate CSCs were transduced with a mixture of viral particles and polybrene with two rounds of infections [147].

### Lentiviral reporter assays

The enhanced d2-eGFP gene (Clontech) was cloned downstream of a LEF-1/TCF-responsive promoter (β-catenin reporter), containing three LEF-1/TCF binding motifs and a TATA box [148]. This cassette was then cloned into a self-inactivating lentiviral vector plasmid (pRRL.sin-18.ppt), and virus was produced as described above. For *in vitro *assays, transduced CSCs were plated at 1,000 cells per well in 96-well plates and treated with EGCG and/or quercetin. After incubation, CSCs were analyzed for GFP expression.

### Caspase-3/7 Assay

Cells (3 × 10^4 ^per well) were seeded in a 96-well plate with 200 μl culture medium. Approximately 16 h later, cells were treated with various doses of EGCG with or without quercetin. Casapse-3/7 activity was measured by a fluorometer as per manufacturer's instructions (Invitrogen).

### Western Blot Analysis

Western blots were performed as we described earlier [149,150]. In brief, cells were lysed in RIPA buffer containing 1 × protease inhibitor cocktail, and protein concentrations were determined using the Bradford assay (Bio-Rad, Philadelphia, PA). Proteins were separated by 12.5% SDS/PAGE and transferred to membranes (Millipore, Bedford, MA) at 55 V for 4 h at 4°C. After blocking in 5% nonfat dry milk in TBS, the membranes were incubated with primary antibodies at 1:1,000 dilution in TBS overnight at 4°C, washed three times with TBS-Tween 20, and then incubated with secondary antibodies conjugated with horseradish peroxidase at 1:5,000 dilution in TBS for 1 hour at room temperature. Membranes were washed again in TBS-Tween 20 for three times at room temperature. Protein bands were visualized on X-ray film using an enhanced chemiluminescence detection system.

### Statistical Analysis

The mean and SD were calculated for each experimental group. Differences between groups were analyzed by one or two way ANOVA, followed by Bonferoni's multiple comparison tests using PRISM statistical analysis software (GrafPad Software, Inc., San Diego, CA). Significant differences among groups were calculated at P < 0.05.

## Abbreviations

ANOVA: Analysis of Variance; BCRP: Breast cancer resistant protein; ESC: Embryonic stem cells; MDR: Multidrug resistance; PI3K: Phosphotidylinositol-3-kinase; PTEN: Phosphatase and Tensin Homolog Deleted on Chromosome 10; SDS-PAGE: Sodium Dodecyl Sulphate-Polyacrylamide Gel Electrophoresis; TBS: Tris Buffer Saline

## Competing interests

The authors declare that they have no competing interests.

## Authors' contributions

ST, CS, DN and DM performed the experiments. SS and RKS designed and wrote the manuscript. All the authors have read and approved the final manuscript.
